# Identification of breast cancer subgroups and immune characterization based on glutamine metabolism-related genes

**DOI:** 10.1186/s12920-023-01792-5

**Published:** 2024-01-10

**Authors:** Hongjing Yu, Junchen Liu

**Affiliations:** 1 Department of Oncology, Jiande Branch, the Second Affiliated Hospital of Zhejiang University School of Medicine, Hangzhou, Zhejiang China; 2 Department of Pharmacy, Jiande Branch, the Second Affiliated Hospital of Zhejiang University School of Medicine, Hangzhou, Zhejiang China

**Keywords:** Glutamine metabolism, Breast cancer, Subgroups, Immune, Gene mutation

## Abstract

Immunotherapy is a promising treatment for breast cancer (BC). However, due to individual differences and tumor heterogeneity, immunotherapy is only applicable to some BC patients. Glutamine metabolism plays a role in inhibiting immunotherapy, but its role in BC is limitedly studied. Therefore, we aimed to identify different BC subgroups based on glutamine metabolism and characterize the features of different subgroups to provide guidance for personalized immunotherapy for BC patients. Using unsupervised clustering analysis, we classified BC patients in The Cancer Genome Atlas (TCGA) with glutamine metabolism-related genes and obtained low-risk (LR) and high-risk (HR) subgroups. Survival analysis revealed that prognosis of LR subgroup was notably better than HR subgroup. Through ssGSEA and CIBERSORT methods, we disclosed that infiltration levels of B cells, Mast cells, T helper cells, and Th2 cells, and Type II IFN Response immune function were notably higher in LR subgroup than in HR subgroup. The Wilcox algorithm comparison denoted that DEPTH of LR subgroup was significantly lower than HR subgroup. The TIDE of LR subgroup was significantly higher than HR subgroup. Functional annotation of differentially expressed genes revealed that channel activity and the Estrogen signaling pathway may be related to BC prognosis. Ten hub genes were selected between the subgroups through the STRING database and Cytoscape, and their correlation with drugs was predicted on the CellMiner website. This study analyzed the immune characteristics of BC subgroups based on glutamine metabolism and provided reference for prognosis prediction and personalized immunotherapy.

**Supplementary Information:**

The online version contains supplementary material available at 10.1186/s12920-023-01792-5.

## Introduction

Breast cancer (BC) is a condition in which breast epithelial cells proliferate out of control while being subjected to several carcinogenic stimuli. Among women, BC accounts for 31% of all new cancer cases and its incidence continues to increase [1]. Although the prognosis of many BC patients may be improved after undergoing surgery, chemotherapy, radiotherapy, and targeted therapy, as a heterogeneous tumor, BC has different tumor morphologies, clinical features, and treatment responses [2, 3]. For example, luminal tumors typically have a favorable clinical outcome, whereas basal-like tumors and HER2-positive tumors have a poor prognosis [4]. Therefore, it is necessary to stratify patients to promote personalized treatment.

Glutamine, the most prevalent and useful amino acid in the body, controls the expression of a wide range of genes involved in metabolism, signal transduction, cell defense, and repair, as well as triggering intracellular signaling pathways [5, 6]. High levels of glutamine in the blood provide carbon and nitrogen sources for cancer cells, driving biosynthesis, energy supplementation, and cellular homeostasis during tumor growth [7]. Studies have found that the loss of glutamine function in triple-negative BC cells causes severe inhibition of tumor growth in vitro and in vivo, while knocking down glutamine has no impact on the growth and metabolite levels of non-triple-negative BC cell lines [8]. Therefore, glutamine metabolism differs between different BC molecular subtypes.

Cancer immunotherapy uses anti-tumor immune responses to activate the host immune system to recognize and eliminate tumor cells [9]. Immunotherapy may be a good treatment option for some BC patients [10, 11]. However, only a few cancer patients can benefit from immunotherapy [12]. Increasing evidence indicates that immune infiltration in tumor microenvironment (TME) is a decisive factor in predicting BC prognosis and immunotherapy response [13]. Late-stage BC patients with high levels of T cells have increased response rates to immunotherapy [14]. Therefore, a comprehensive evaluation of tumor immune cell infiltration is a reliable and effective method to assess BC patients’ sensitivity to immunotherapy. Based on glutamine metabolism, clustering analysis of BC patients, and understanding the immune characteristics of different patient types can promote personalized treatment and increase patients’ benefits from immunotherapy.

In this study, we used genomic and transcriptomic data from 1,226 BC samples from The Cancer Genome Atlas (TCGA) dataset, as well as glutamine metabolism-related genes, to classify patients. We then analyzed the immune landscape of different subgroups of patients using the single sample GSEA (ssGSEA) algorithm and CIBERSORT algorithm. Finally, we analyzed the biological functional differences, hub gene selection, and drug sensitivity prediction between different subgroups. Our results revealed the potential connection between TME and immunotherapy for different BC subgroups based on glutamine metabolism-related genes, which can help tailor immunotherapy strategies for BC patients.

## Materials and methods

### Data source

mRNA expression data for BC was available for download at TCGA (https://portal.gdc.cancer.gov/), involving 113 normal and 1113 BC samples, along with corresponding clinical data. The samples included Luminal A, Luminal B, HER-2 overexpressing, Basal-like, and Normal-like subtypes. GSE21653 was downloaded from the GEO database (https://www.ncbi.nlm.nih.gov/geo/) as a validation set, and the dataset comprised gene expression profile data from 266 BC patients. A total of 79 glutamine metabolism-related genes were obtained from MSigDB (http://www.gsea-msigdb.org/gsea/msigdb/index.jsp). These genes were involved in biological processes such as glutamine synthesis, degradation, transport, and regulation (Table S1).

### Unsupervised clustering based on glutamine metabolism-related genes

Unsupervised clustering analysis is a common data mining technique that uses hierarchical consensus clustering to analyze patient clustering with expression of glutamine metabolism-related genes. The optimal number of clusters was determined using consensus clustering algorithms, and to ensure stability of results, original data was subjected to 1000 random resampling and clustering analyses to obtain a stable clustering result. R package “ConsensusClusterPlus” [15] was utilized to conduct above steps. R package “survival” (https://github.com/therneau/survival) was utilized to study the differences in survival status between BC subgroups.

### TME landscape analysis

To assess the tumor microenvironment of each sample in the subgroups, the R package “estimate” [16] was utilized. This package employed single-sample gene set enrichment analysis (ssGSEA) to compute stromal score, immune score, ESTIMATE score, and tumor purity. Wilcoxon test was then used for comparisons, and these comparative results were visually represented more effectively through violin plots. R package “pheatmap” [17] was utilized to visualize anti-tumor immune enrichment status of the two subgroups. The CIBERSORT method was utilized to reveal immune infiltration levels and Wilcox test was used to compare between two subgroups. Box plots were used to make the results clearer and easier to understand.

### Intra-tumor heterogeneity (ITH) and Tumor immune dysfunction and exclusion (TIDE) analysis

ITH refers to the differences between tumor cells and is closely linked with tumor progression, dismal prognosis, immune suppression, genomic instability, and treatment resistance. Therefore, evaluating ITH levels is important for tumor prognosis and the success of immunotherapy, and is one of the current hotspots in tumor research [18]. To accurately evaluate ITH levels, many methods and algorithms have been proposed in recent years. Among them, Deviating Gene Expression Profiling Tumor Heterogeneity (DEPTH) algorithm based on mRNA levels has been widely used to evaluate ITH levels. Wilcox test was utilized to analyze differences in DEPTH scores between subgroups. Violin plots were generated. TIDE algorithm was employed to score the high-risk (HR) and low-risk (LR) groups, evaluating the potential responsiveness to immunotherapy. Wilcoxon test was conducted on the TIDE scores of the two subgroups to determine if there was a significant difference in TIDE scores.

### Tumor mutation analysis between subgroups

SNV mutation data for BC was collected, and the top 30 most frequent mutation genes were selected from each subgroup and compiled. Mutation data of top 30 genes in two subgroups was statistically analyzed and organized. The selected mutated genes were organized and analyzed, including calculation of their mutation frequencies and the distribution of different mutation types. We conducted a literature validation for the chosen top 30 most frequent mutated genes to ensure their relevance in BC research and excluded genes unrelated to the study objectives. The R package “GenVisR” [19] was downloaded and installed, and the waterfall plot was generated using the functions in the “GenVisR” package to display the frequency and type of these mutation genes more clearly.

### Functional enrichment analysis of differentially expressed genes (DEGs) between subgroups

The “edgeR” package [20] in R was used to perform differential analysis between the two subgroups, and DEGs were selected according to criteria of FDR < 0.05 and|logFC|>1.Gene Ontology(GO)enrichment analysis is employed to detect the enrichment patterns of genes in biological processes, molecular functions, and cellular components [21]. Kyoto Encyclopedia of Genes and Genomes (KEGG) is a comprehensive genomic database that combines genomic information with biological processes such as biochemical reactions, metabolic pathways, and cellular signaling, providing researchers with a comprehensive genomic research platform [22, 23]. The “clusterprofiler” package was utilized for GO and KEGG enrichment analyses of selected genes, with *P* < 0.05 meant statistically significant. This step can provide important clues for biological research by gaining a deeper understanding of the functional and biological process differences between the two subgroups [24].

### PPI network construction and hub gene selection between subgroups

DEGs of BC were input into STRING to build PPI network and further study mechanisms of gene function and disease occurrence. In building the PPI network, interaction relationships with confidence scores higher than 0.9 were selected. After obtaining the PPI network data, Cytoscape software was used for visualization and analysis. CytoHubba plugin in Cytoscape was utilized to calculate hub genes in PPI network, which were crucial for maintaining network stability and function between subgroups.

### Drug sensitivity analysis of hub genes between subgroups

CellMiner (https://discover.nci.nih.gov/cellminer/) [25] is a public database that contains genomic, drug sensitivity, and related data for varying human cancer cell lines. We utilized this website to explore relationship between genes and drugs and predict targeted drugs suitable for different subgroups of patients based on the hub genes.

## Results

### Assignment of BC patients into LR and HR subgroups based on glutamine metabolism-related genes

**Fig. 1:**
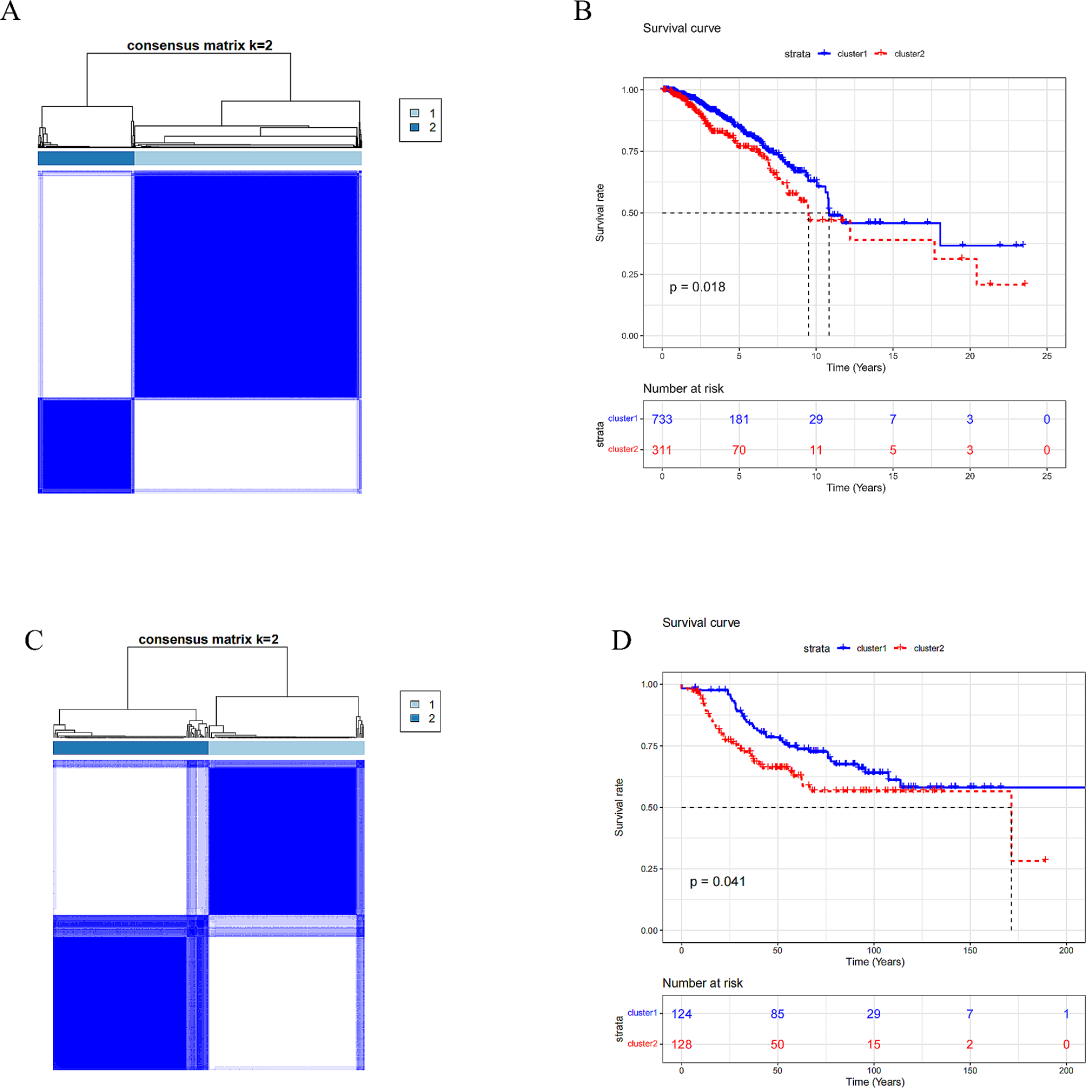
Subgroup identification and survival analysis of BC patients in the TCGA and GEO datasets. (**A**) The optimal clustering number in the TCGA dataset was determined to be K = 2, the greater the distance between two samples, the less similar they are, and the smaller the distance, the more similar they are. (**B**) Survival analysis of BC LR and HR subgroups in the TCGA dataset. The blue curve represents the LR subgroup, the red curve represents the HR subgroup, the X-axis represents survival time counted in years, and the Y-axis represents the survival probability of the corresponding subgroup. The dotted line represents the median survival time and survival probability of the corresponding subtype. (**C**) The optimal clustering number in the GEO dataset. (**D**) Survival analysis of LR and HR subgroups of BC in the GEO dataset

Unsupervised clustering analysis was performed on patients with a survival time greater than 30 days according to expression of glutamine metabolism-related genes. The optimal number of clusters was determined to be 2 (k = 2) using consensus clustering algorithms, and the samples were divided into two subgroups, with 733 patient samples in subgroup 1 and 311 patient samples in subgroup 2 (Fig. 1A). Survival analysis was completed on two subgroups. Survival of subgroup 1 was substantially better than subgroup 2 (Fig. 1B). To further validate the impact of glutamine metabolism-related genes on the survival of BC patients, we conducted consensus clustering again in the GEO dataset. The clustering results revealed that all samples could be divided into two subgroups, and the survival of these two subgroups still exhibited significant differences (*P* < 0.05) (Fig. 1C-D). Therefore, we defined Subgroup 1 in the TCGA dataset as LR subgroup and Subgroup 2 as HR subgroup. Subsequent analyses focused on exploring the characteristics of these two subgroups.

### TME features in different subgroups

**Fig. 2:**
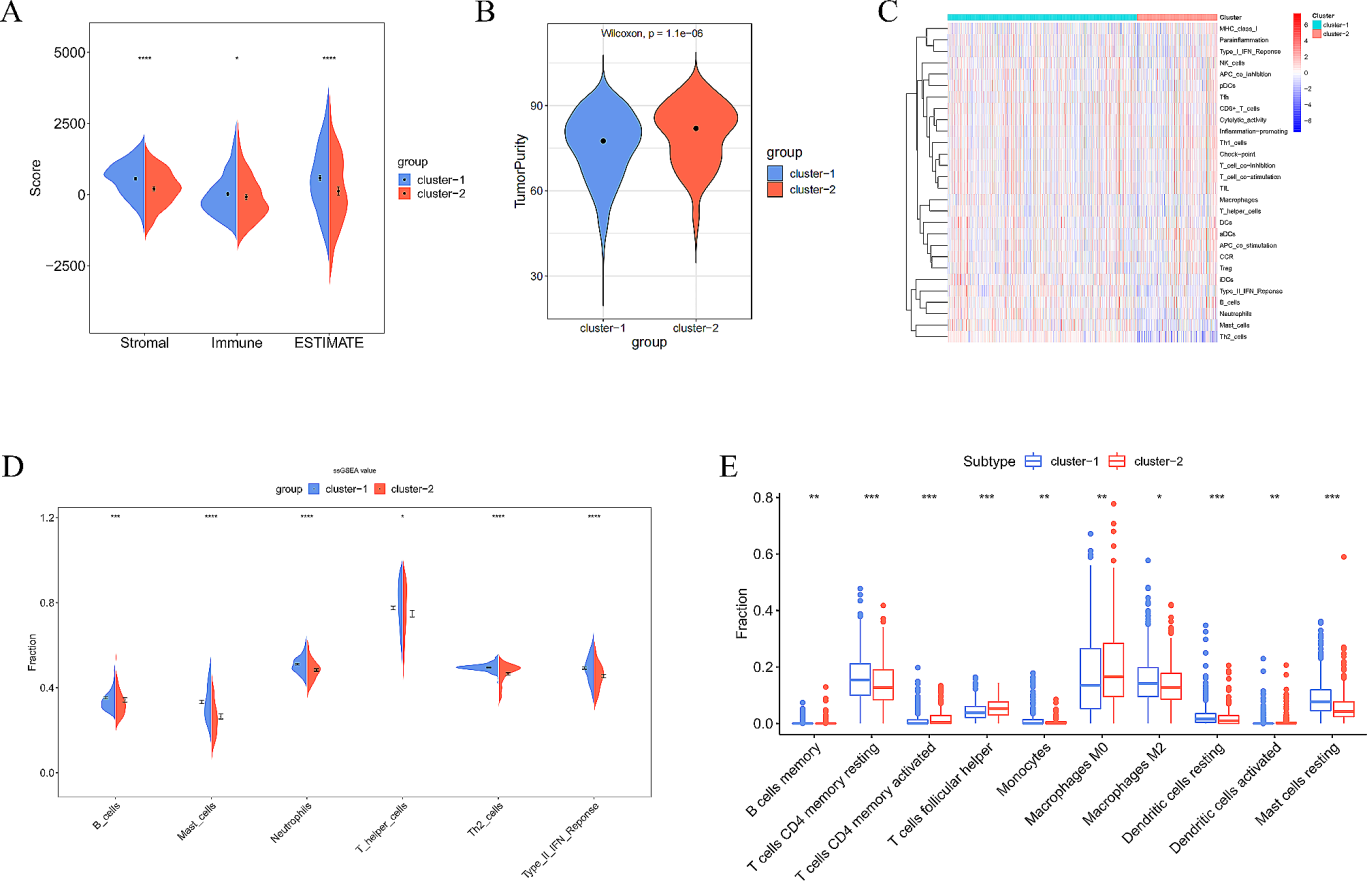
Characteristics of TME in different subgroups. (**A**) Immune-related score analysis between BC subgroups. (**B**) Analysis of tumor purity between BC subgroups. (**C**) Analysis of the correlation between BC subgroups and immune cell components using the ssGSEA method. (**D**) Analysis of immune-related cell expression and immune function between BC subgroups using the ssGSEA method. (**E**) Analysis of immune-related cell expression between BC subgroups using the CIBERSORT method. (* means *P* < 0.05; ** means *P* < 0.01; *** means *P* < 0.001)

Differential analysis was performed on the stromal cell component, immune cell component, and ESTIMATE score of BC samples in TCGA dataset. Stromal, immune, and ESTIMATE scores of LR subgroup were significantly higher than those of HR subgroup (*P* < 0.05)(Fig. 2A). The tumor purity of LR subgroup was significantly lower than HR subgroup (*P* < 0.05) (Fig. 2B). Immune-related cell infiltration levels of each BC sample were evaluated by ssGSEA, and results showed that infiltration levels of B cells, Mast cells, T helper cells, Th2 cells, and Type II IFN Response immune function were notably higher in LR subgroup than in HR subgroup (*P* < 0.05) (Fig. 2C-D). To gain a more accurate understanding of differences in immune levels between subgroups, we further quantified immune cell infiltration level of tumors by CIBERSORT method. Most immune cell infiltration levels were substantially higher in LR subgroup than in HR subgroup (*P* < 0.05) (Fig. 2E). This indicated that LR subgroup exhibited “hot tumor” features, with higher levels of immune cell infiltration, which may assist in immunotherapy [26].

### Prediction of the response of BC patients to immunotherapy

**Fig. 3:**
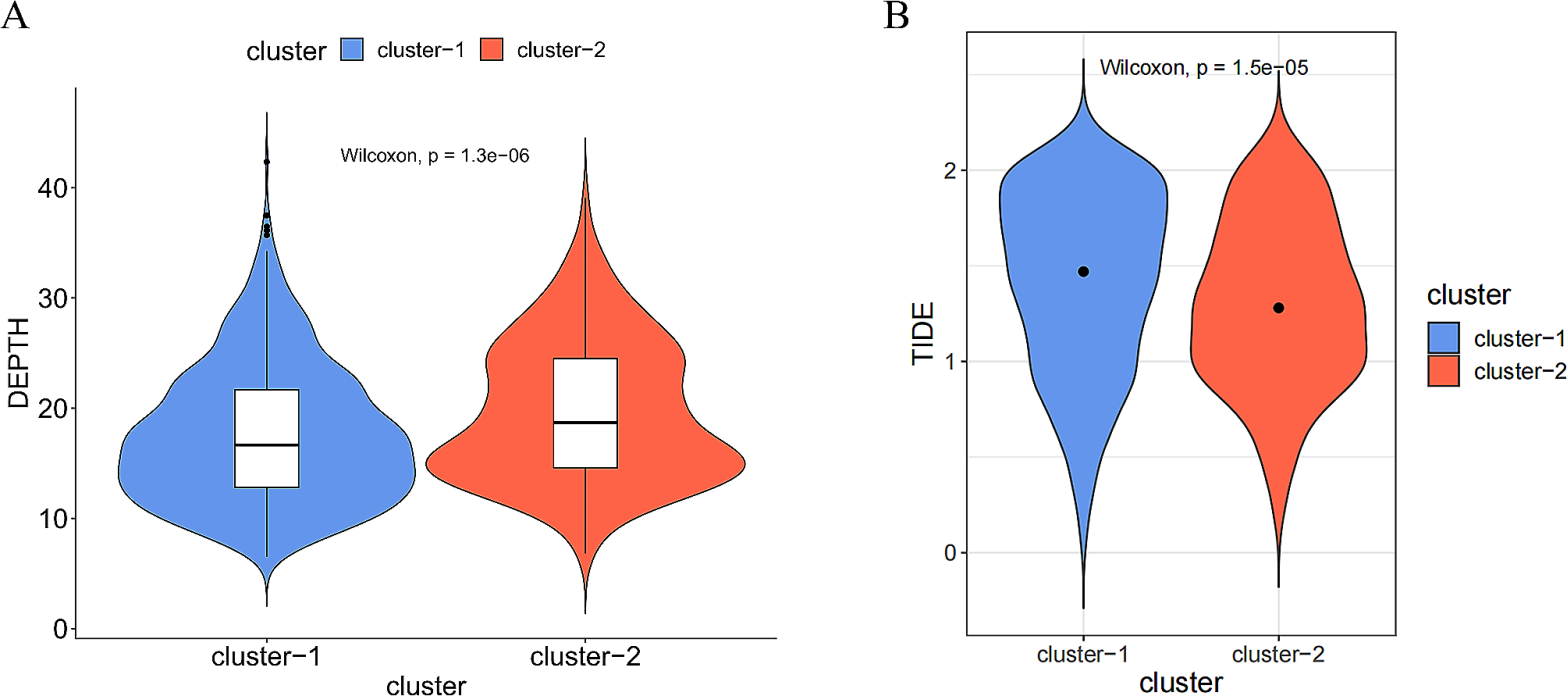
Analysis of ITH and TIDE between BC subgroups. (**A**) DEPTH score analysis between BC subgroups. (**B**) TIDE score analysis between BC subgroups

ITH is a marker of tumor development and evolution and has potential clinical significance, with lower ITH levels indicating greater suitability for immunotherapy [18]. To investigate the ITH and TIDE features of the two BC subgroups, we calculated the DEPTH and TIDE scores of both subgroups and performed Wilcox tests. DEPTH score of the LR subgroup was significantly lower than HR subgroup (Fig. 3A). TIDE scores of the LR subgroup were significantly higher than HR subgroup (*P* < 0.05) (Fig. 3B). This suggested that patients in the HR subgroup of BC exhibited higher resistance to immunotherapy and a higher likelihood of immune escape. Conversely, patients in the LR subgroup may be more suitable candidates for immunotherapy.

### Tumor mutation burden features in different subgroups

**Fig. 4:**
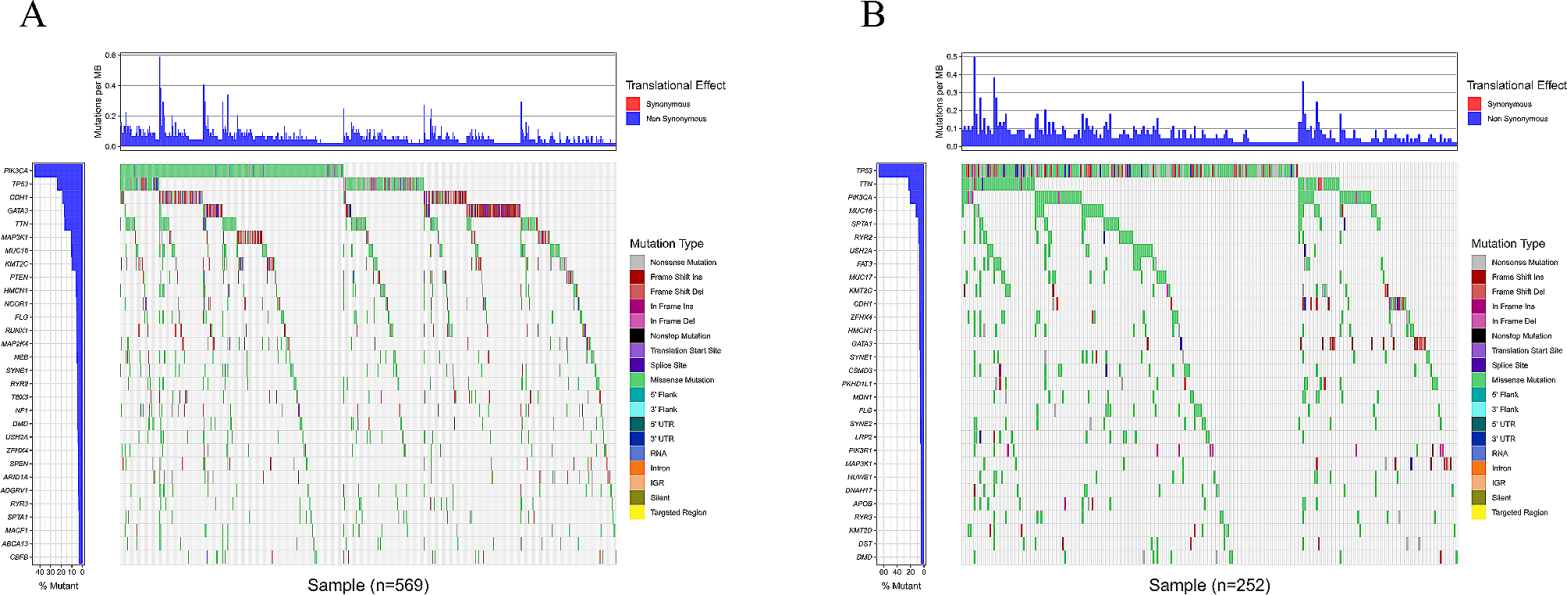
Gene mutation results of BC LR and HR subgroups. (**A**) Mutation status of the top 30 genes with the highest mutation frequency in the LR subgroup. (**B**) Mutation status of the top 30 genes with the highest mutation frequency in the HR subgroup. The mutation frequency of each gene is displayed by the bar chart on the left, and the number of mutation loads is displayed in the bar chart above the legend. Different colors in the legend correspond to different mutation types

We collected SNV mutation data for both BC subgroups and analyzed the top 30 genes with high mutation frequencies in LR and HR subgroups. We generated a waterfall plot to display distribution of mutation frequencies for these genes. In LR subgroup, PIK3CA, TP53, CDH1, GATA3, and TTN were found to have higher mutation frequencies (Fig. 4A), while in HR subgroup, TP53, TTN, PIK3CA, MUC16, and SPTA1 had higher mutation frequencies (Fig. 4B).

### Identification and functional annotation of DEGs in LR and HR subgroups of BC

**Fig. 5:**
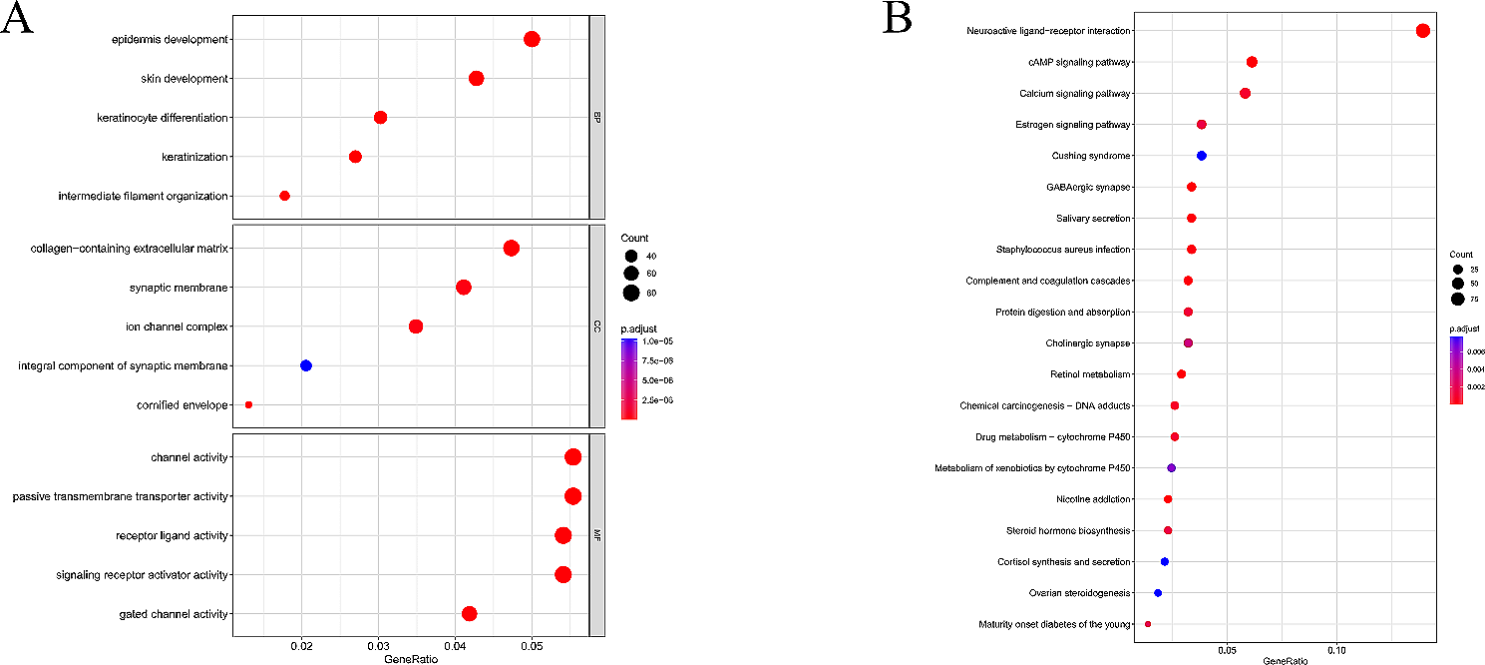
GO and KEGG enrichment analyses of DEGs in BC LR and HR subgroups. (**A**) GO enrichment analysis results of DEGs. (**B**) KEGG enrichment analysis results of DEGs. Each bubble represents a GO function or KEGG pathway, and the size of the bubble reflects the number of genes enriched in the corresponding function or pathway. The color of the bubble represents the significance of the *P* value, with red to dark blue representing low to high *P* values

Given significant survival differences between LR and HR subgroups, we dissected molecular differences at molecular level, aiming to explore mechanisms of survival differences. Analysis of the genes in both subgroups revealed that 1755 genes were differentially expressed between LR and HR subgroups (Table S2). GO and KEGG enrichment analyses were conducted on DEGs of both subgroups. GO analysis results showed that enrichment of these genes exhibited mainly in biological functions such as epidermis development, collagen-containing extracellular matrix, channel activity, and passive transmembrane transporter activity (Fig. 5A). The KEGG analysis showed enrichment of DEGs in pathways such as Cushing syndrome, Estrogen signaling pathway, Calcium signaling pathway, cAMP signaling pathway, and Neuroactive ligand-receptor interaction (Fig. 5B). Therefore, channel activity, passive transmembrane transporter activity, and Estrogen signaling pathway may be the key factors contributing to the survival differences between LR and HR subgroups.

### Screening of hub genes in LR and HR subgroups of BC

**Fig. 6:**
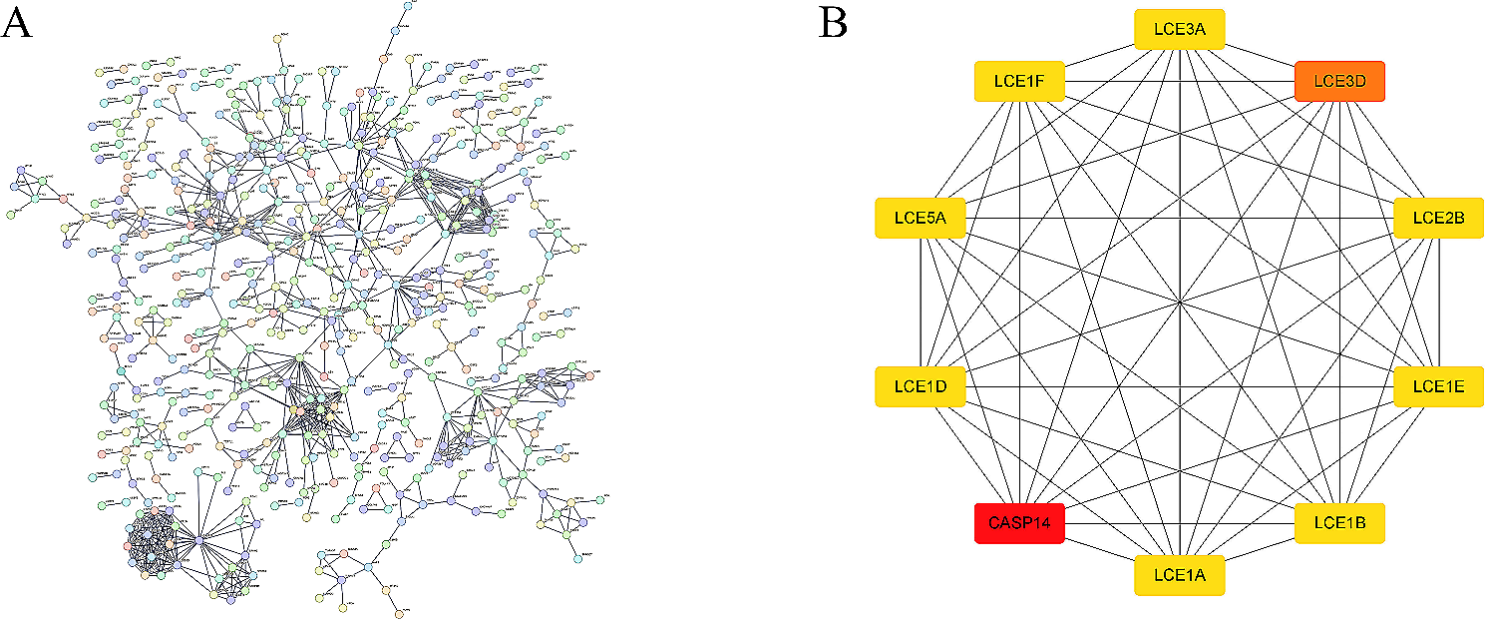
Identification of hub genes for BC LR and HR subgroups. (**A**) PPI network visualization of DEGs between subgroups. (**B**) The top 10 genes with the highest connectivity were selected as hub genes using the MCC method of the cytoHubba plugin

DEGs between subgroups were utilized to construct a PPI network, and selecting high-confidence interaction relationships could improve the reliability and accuracy of the network. Therefore, we selected interaction relationships with confidence scores higher than 0.9 from STRING to build PPI network, which had 1,716 nodes and 919 edges with an average node degree of 1.07. This PPI network was used to reveal interactions between DEGs (Fig. 6A). We input network data into Cytoscape and implemented CytoHubba plugin to screen the top 10 hub genes in the network, which were CASP14, LCE3D, LCE1D, LCE5A, LCE1F, LCE1A, LCE3A, LCE1B, LCE1E, and LCE2B. The positions and interaction patterns of these genes in PPI network may be key in distinguishing biological processes and functions between different subgroups (Fig. 6B).

### Drug sensitivity prediction of hub genes between subgroups of BC

**Fig. 7:**
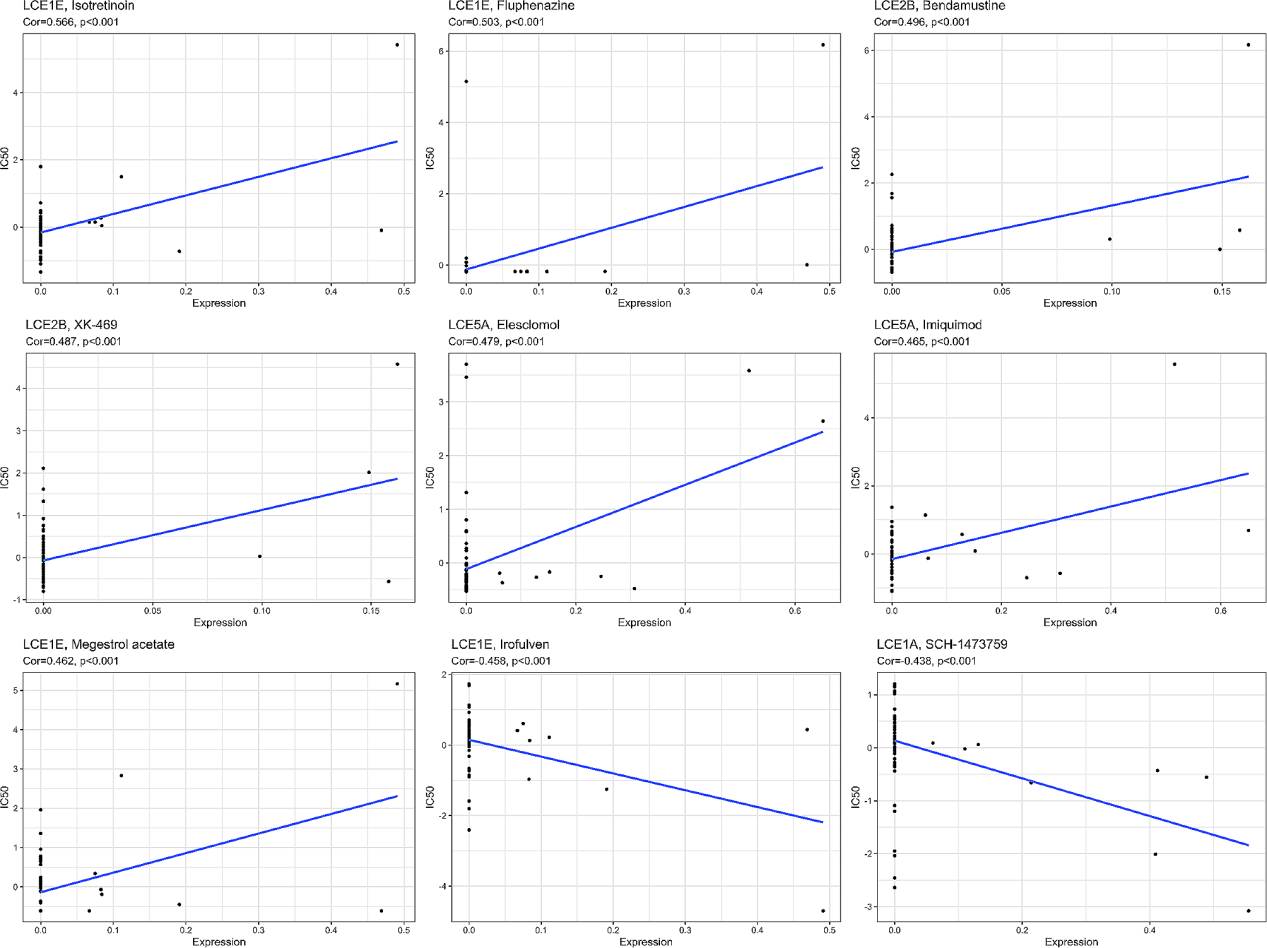
Predicted results of hub genes in BC LR and HR subgroups in CellMiner (IC_50_ refers to the drug concentration required to reduce the number of surviving cells by half after drug treatment. The lower the IC_50_, the more sensitive to the drug, and the stronger the inhibitory effect on tumor cells. The correlation coefficient Cor between 0.1–0.3 indicates weak correlation, 0.3–0.5 indicates moderate correlation, and 0.5-1.0 indicates strong correlation.)

We utilized CellMiner database to predict correlation of hub genes with drug sensitivity and found that LCE1E was positively correlated with the IC_50_ values of Isotretinoin, Fluphenazine, and Megestrol acetate (Cor > 0.4, *P* < 0.001) and negatively correlated with IC_50_ value of Irofulven (Cor > 0.4, *P* < 0.001). LCE2B was positively correlated with the IC_50_ values of Bendamustine and XK-469 (Cor > 0.4, *P* < 0.001), LCE5A was positively correlated with IC_50_ values of Elesclomol and Imiquimod (Cor > 0.4, *P* < 0.001), and LCE1A was negatively correlated with IC_50_ value of SCH-1,473,759 (Cor > 0.4, *P* < 0.001) (Fig. 7).

### Discussion

In this study, we divided BC into LR and HR subgroups based on glutamine metabolism-related genes. By comparing survival rates, immune-related cells, and immune function indicators between two subgroups, we disclosed that patients in LR subgroup had better prognostic performance and higher immune function levels, while patients in HR subgroup had higher DEPTH scores. In LR subgroup, the PIK3CA gene had a relatively high mutation frequency, while in HR subgroup, TP53 and TTN genes had higher mutation frequencies. Pathway analysis showed that DEGs in both subgroups were mainly enriched in channel activity biological function and Estrogen signaling pathway. We investigated correlation of prognostic-related genes with drugs. This study provided new subgroup characteristics for BC and could predict prognosis and provide personalized immune therapy recommendations based on these characteristics.

We disclosed that LR subgroup had better prognosis and noticeably higher infiltration levels of various immune-related cells compared to the HR subgroup. B cells are a pivotal component of immune system and can affect tumor development and prognostic outcomes through various pathways [27]. You et al. [28] found that higher levels of B cells are notably associated with better survival rates in BC patients, and tumor-infiltrating B cells were a biomarker of good prognosis in BC patients. The role of mast cells in cancer is controversial, and their beneficial or harmful effects on tumors depend on the tumor type and their location within the tumor [29]. Some studies have indicated that mast cells may be implicated in better prognosis in HR-positive BC patients [30]. The number of T helper cells within the tumor is positively correlated with advanced tumor stage, tumor volume, and positive tumor metastasis, and is associated with dismal prognosis of BC patients [31], which is opposite to the results of this study. However, Matsumoto et al. [32] reported that high levels of T helper cells indicate good prognosis in triple-negative BC patients, indicating that T helper cells may have different prognostic outcomes in different subtypes of BC patients. Th2 cells are a subtype of T helper cells that can directly block spontaneous BC development by facilitating terminal differentiation of cancer cells [33]. Several studies have suggested that M2 macrophages in BC may be associated with the malignancy of tumors and adverse prognosis [34, 35]. In contrast to other research, this study observed a significant increase in M2 macrophages in the LR subgroup with a favorable prognosis. M2 macrophages are typically linked to anti-inflammatory responses and the attenuation of host immune reactions [36, 37]. In the LR subgroup, the immune system may respond more actively, leading to an increased presence of M2 macrophages. Additionally, BC tissues often harbor lesions [38], and M2 macrophages play a role in phagocytosing and clearing dead cells, cell fragments, and other debris, promoting wound healing and maintaining a favorable tissue microenvironment [39]. This could be a contributing factor to the significant increase observed in the LR subgroup. Based on the above studies, we found that subgroup classification based on glutamine metabolism-related genes can predict prognoses of BC patients, and patients with good prognosis have characteristics of high levels of infiltrating B cells and mast cells.

Immunotherapy has revolutionized cancer treatment and different types of cancer patients may benefit from different treatment modalities [40]. The DEPTH score is an indicator for evaluating tumor prognosis and immune therapy response. Specifically, a lower DEPTH score generally indicates better prognosis and a higher response to immune therapy [41]. Our study found that DEPTH score of LR subgroup was remarkably lower than that of HR subgroup. Song et al. [42] found in their study of pan-cancer that a high DEPTH2 score is implicated in poor survival rates in ten cancer types, including BC, and that a high DEPTH2 score may reduce the response to immune therapy. This further underscores the importance of the DEPTH score in prognosis and immunotherapy response, which is comparable to our findings. Patients in LR subgroup of BC may be more suitable for receiving immunotherapy. Gene mutations can also serve as valuable biomarkers for predicting immunotherapy response [43]. PIK3CA has a relatively high mutation frequency in LR subgroup, while TP53 and TTN have higher mutation frequencies in HR subgroup. PIK3CA mutations in BC are highly heterogeneous, and better characterization of PIK3CA mutations can help determine treatment methods [44]. TP53 mutations can promote immune activity in BC patients, and their mutation status may be a biomarker for predicting immunotherapy response in BC patients [45]. Pan et al. [46] disclosed that TP53/PIK3CA/ATM mutations can predict response to immunotherapy in bladder cancer patients. In our study, highly mutated PIK3CA and high immune cell infiltration status may be more favorable for immunotherapy response in LR subgroup of BC. Additionally, we conducted hub gene screening for two subgroups. Through the prediction of these hub genes, we identified potential drugs for the treatment of BC, such as Fluphenazine, Megestrol acetate, and Bendamustine, aiming to provide insights into BC treatment. Previous studies have indicated that Fluphenazine can effectively inhibit tumor growth and metastasis in a triple-negative BC mouse model [47]. Megestrol acetate, a synthetic progestin used in BC treatment, has demonstrated therapeutic effects in hormone-sensitive advanced BC patients in clinical trials [48, 49]. A clinical trial has shown that the combination of Bendamustine with Capecitabine is effective in treating HER2-negative metastatic BC patients [50]. These drugs have shown potential effects in BC treatment in clinical research. Therefore, our research results hold promise in providing beneficial clues for personalized treatment and laying the theoretical foundation for future in-depth research and drug development.

DEGs were mainly enriched in channel activity biological function and Estrogen signaling pathway. Xu et al. [51] found that the calcium channel TRPV6 drives BC invasion and metastasis through NFATC2IP and is implicated in dismal prognosis in BC. Other studies have found that overexpression of the ion channel TRPM7 may be implicated in dismal prognosis in BC patients [52]. Estrogen helps regulate the differentiation and proliferation of normal mammary epithelial cells, and its overexpression is linked with elevated risk of BC [53]. Estrogen mainly promotes BC cell growth by activating estrogen receptors, and estrogen is pivotal in progression from primary BC to metastatic BC [54]. Zhuang et al. [55] found that TRIM3 promotes BC cell migration and proliferation by promoting estrogen signaling. We speculated that channel activity biological function and Estrogen signaling pathway may be critical in BC prognosis.

In conclusion, this work assigned BC patients into LR and HR subgroups. Compared to patients in HR subgroup, patients in LR subgroup had good prognosis and high immune cell infiltration. This superior immune status may help patients in LR subgroup achieve favorable therapeutic efficacy after receiving immune therapy, and our study results may provide insights into BC classification and treatment strategies. However, our study still has certain limitations, as we only made predictions based on databases and lacked experimental and clinical data validation.

### Electronic supplementary material

Below is the link to the electronic supplementary material.

Supplementary Material 1

Supplementary Material 2

## Data Availability

The data that support the findings of this study are available from TCGA(https://portal.gdc.cancer.gov/), GEO database (https://www.ncbi.nlm.nih.gov/geo/), MSigDB (http://www.gsea-msigdb.org/gsea/msigdb/index.jsp).
